# Regulatory mechanisms of Traditional Chinese Medicine on gut microbiota in diabetic microvascular complications

**DOI:** 10.1186/s13020-026-01374-4

**Published:** 2026-03-18

**Authors:** Yiyang Peng, Chenyao Sun, Zheng Wang, Xiaohui Fan

**Affiliations:** 1 College of Pharmaceutical Sciences, Zhejiang University, Hangzhou, 310058 China; 2 State Key Laboratory of Chinese Medicine Modernization, Innovation Center of Yangtze River Delta, Zhejiang University, Jiaxing, 314100 China; 3 Zhejiang Key Laboratory of Chinese Medicine Modernization, Innovation Center of Yangtze River Delta, Zhejiang University, Jiaxing, 314100 China

**Keywords:** Traditional Chinese Medicine, Diabetic microvascular complications, Gut microbiota

## Abstract

Diabetic microvascular complications are the most common and serious late-stage problems with diabetes patient, while existing antidiabetic treatments still have limited effects in preventing or reversing microvascular damage. Traditional Chinese Medicine (TCM) has been applied in clinical practice for centuries, showing excellent therapeutic effects in alleviating diabetic microvascular complications. Recently, the regulatory effects of gut microbiota, especially concepts such as the “gut-retina axis” and “gut-kidney axis” have been proposed and supported by accumulating experiment, which shows the importance of gut microbiota in microvascular injury. This review focuses on how TCM-gut microbiota interactions contribute to the prevention and treatment of diabetic microvascular complications, reviewing and organizing the latest evidence from experimental and clinical research. We further summarize current limitations in the field and discuss future direction to better uncover the mechanisms of TCM-microbiota interactions.

## Introduction

Diabetes, a chronic metabolic syndrome, is featured by prolonged hyperglycemia [1, 2]. International Diabetes Federation (IDF) reported that there are around 537 million adults globally are suffering from diabetes, and this number could climb to 783 million by 2045 [3]. Long-term chronic hyperglycemia can cause a damage of the structure and function of microvasculature throughout the whole body, eventually leading to the development of diabetic microvascular complications [4–6]. Diabetic microangiopathy is the earliest and most common complication of diabetes, characterized by capillary damage [7, 8]. Its main pathological features include microcirculatory dysfunction [9], thickening of the basement membrane [10], formation of microaneurysms [11], microthrombosis and capillary occlusion [12], as well as neovascularization [12]. Clinically, it presents as diabetic retinopathy (DR) [13], diabetic kidney disease (DKD) [14], and diabetic peripheral neuropathy (DPN) [15].The pathogenesis of these complications is complex, involving multiple metabolic disturbances and activation of inflammatory signaling pathways, including advanced glycation end-products (AGEs) accumulation [15], the polyol pathway activation [16], and protein kinase C (PKC) signal dysregulation [17].The treatment of diabetic microvascular complications primarily involves comprehensive interventions such as glycemic control [18], blood pressure reduction [8], lipid regulation [19], anti-inflammatory therapy [20], and antioxidant strategies [21]. Currently, widely used antidiabetic drugs such as Metformin [22], GLP-1 receptor agonists [23], and SGLT2 inhibitors [24] are widely used as antidiabetic drugs currently since they have shown significant efficacy in improving glycemic control. However, their direct protective effects on microvascular complications remain limited. The deficiencies in treatment highlights an urgent need to explore novel regulatory mechanisms and intervention strategies beyond conventional glucose-lowering agents. In this context, the human gut microbiota has gained growing attention as a potential key player participant in diabetes and its complications.

Human gut microbiota, known as the largest and most diverse microbial community in the body, has been a central topic in microbiome studies for a long time [25, 26]. It has been clearly demonstrated that hundreds of microbial species coexist in the gut and affect both physiological and pathological processes in the host. In recent years, breakthroughs in gene sequencing and multi-omics technologies [27] have further revealed the potential regulatory effects of the gut microbiota on diabetic microvascular complications [28–30]. The hypotheses of the “gut-kidney axis” [31] and “gut-retina axis” [32] has been proposed, which further support the significant involvement of gut microbiota in the pathogenesis of DKD [33] and DR [34]. Gut dysbiosis influences diabetic microvascular injury through several mechanisms, including modulation of systemic inflammation, production of microbial metabolites such as short-chain fatty acids, regulation of oxidative stress, and alteration of intestinal barrier integrity, all of which affect vascular function. Increasing evidence has connected gut dysbiosis with the progression of diabetic microangiopathy, while also recognizing its potential for therapeutic intervention. This offers new perspectives and strategies for the prevention and treatment of diabetic complications such as DR.

Traditional Chinese Medicine (TCM) has shown great potential in the prevention and treatment of diabetes and its microvascular complications [35, 36], owing to its multi-target and multi-pathway regulatory effects [37, 38]. Modern pharmacological studies have revealed that TCM can directly improve glucose and lipid metabolism [39], exert antioxidant effects [40], inhibit inflammatory responses [41], and prevent vascular damage [42]. Additionally, TCM regulates the gut microbiota [43] to influence the metabolic environment, thereby delaying the development of diabetic microvascular complications. To better understand these effects, it is important to identify the major bioactive constituents in TCM and their modes of action on the gut microbiota. Bioactive constituents of TCM can be broadly categorized into macromolecular components, small-molecule compounds, and plant-derived exosome-like nanovesicles (PELNs). Macromolecules such as polysaccharides and saponins generally escape digestion in the small intestine and undergo microbial fermentation in the colon, thereby reshaping community structure and metabolic outputs. Small molecules including flavonoids and alkaloids are more readily absorbed and can directly influence gut microbiota by suppressing pathogenic species or promoting beneficial taxa, while simultaneously acting on host pathways. More recently, PELNs have been shown to transfer lipids, metabolites, and RNAs to intestinal microbes or host cells, mediating cross-kingdom regulation of microbial function and barrier integrity. Together, these bioactive categories illustrate the diverse ways in which TCM components modulate the gut microbiota, laying the foundation for their therapeutic potential in metabolic and vascular complications.

**Fig. 1:**
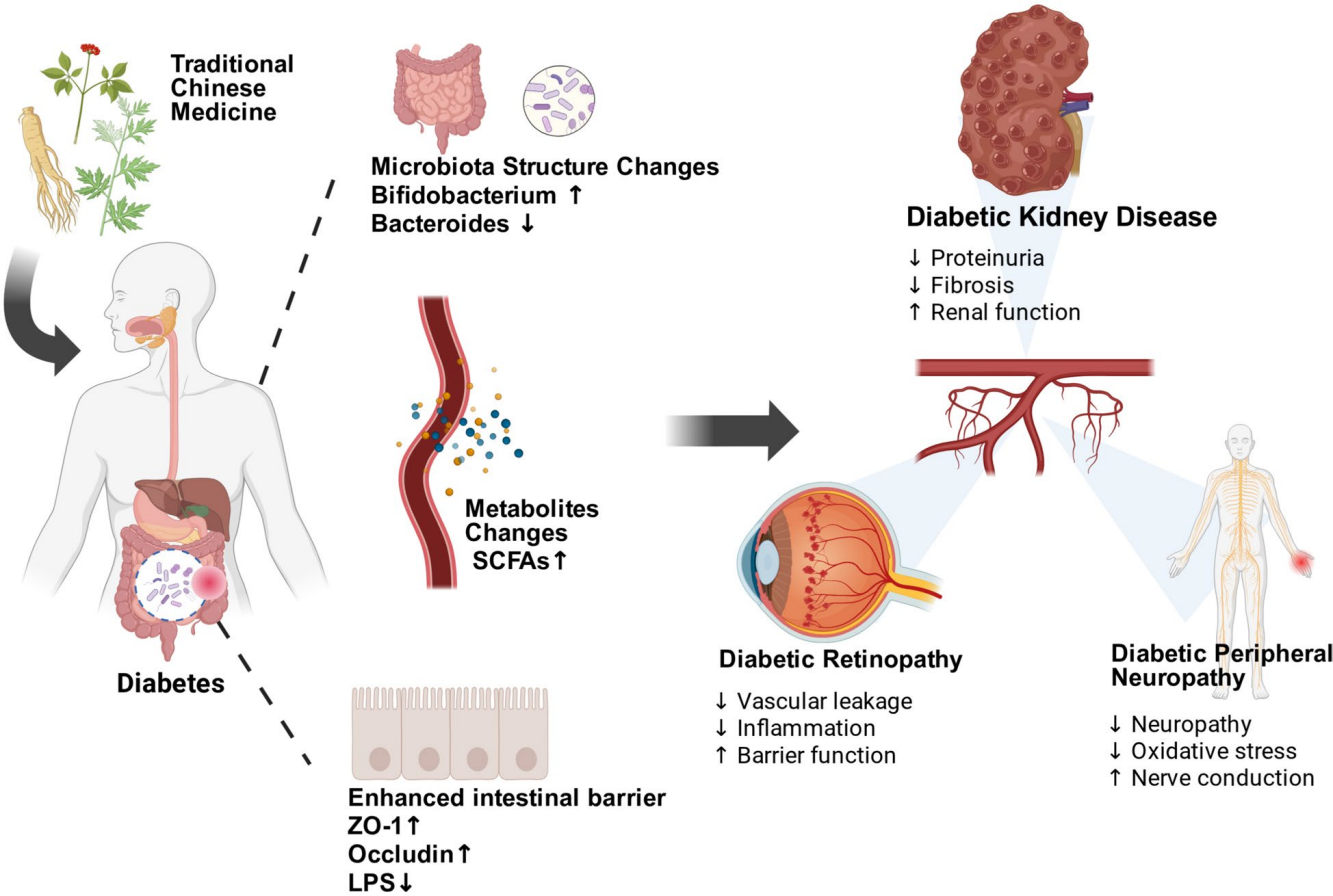
TCM ameliorates diabetic microvascular complications by modulating the gut microbiota, enhancing intestinal barrier integrity, and regulating metabolic

Despite increasing evidence, a systematic understanding of how TCM-microbiota interactions contribute to the prevention and treatment of diabetic microvascular complications is still lacking. Therefore, this review therefore provides recent progress in the investigations of TCM-mediated regulation of gut microbiota in the treatment of diabetic microvascular complications, with a focus on mechanisms involving metabolic reprogramming and vascular protection. By highlighting the essential effects of gut microbiota in the pathophysiology of diabetic microvascular disease, this review demonstrated how TCM interventions reshape microbial composition and metabolite profiles, restore intestinal barrier integrity, reduce inflammation, and modulate the gut-vascular axis. Moreover, the limitations in current research, including insufficient validation of microbiota function, unclear enzymatic and molecular targets, and the challenges of clinical translation, are also discussed. This work provides valuable reference for future studies on the role of TCM-gut microbiota interactions in combating diabetic microvascular complications. (Fig. 1).

## Role of gut microbiota in the development and progression of diabetic complications

### Pathogenesis of diabetic complications

Diabetic microvascular complications primarily include DR, DKD, and DPN. The pathogenesis of these complications involves a complex pathological network composed of multiple mechanisms, including metabolic imbalance, oxidative stress, chronic inflammation, and hemodynamic abnormalities [44]. These factors can trigger a series of pathological changes through pathways such as the polyol pathway, accumulation of AGEs, and activation of PKC pathway. In the polyol pathway, hyperglycemia enhances the activity of aldose reductase (AR), which converts glucose into sorbitol. This leads to intracellular osmotic imbalance, cellular edema and apoptosis, while simultaneously consuming NADPH and weakening the cell's antioxidant defense capacity [45]. The accelerated formation of AGEs not only directly damages the vascular basement membrane and increases its permeability, but additionally triggers the nuclear factor-κB (NF-κB) pathway via the receptor for AGEs (RAGE), enhancing the secretion of proinflammatory cytokines such as TNF-α and IL-6, thereby intensifying the inflammatory response [46]. At the same time, hyperglycemia elevates diacylglycerol levels, activates the PKC pathway, increases VEGF expression, and induces upregulation of TGF-β, further advancing the progression of diabetic microvascular complications [47].

Although hyperglycemia-driven metabolic and signaling abnormalities form the core mechanisms of diabetic complications, emerging evidence suggests that systemic factors beyond blood glucose, particularly the gut microbiota, also play a significant role in modulating disease progression.

### Gut microbiota dysbiosis and diabetic microvascular complications

**Fig. 2:**
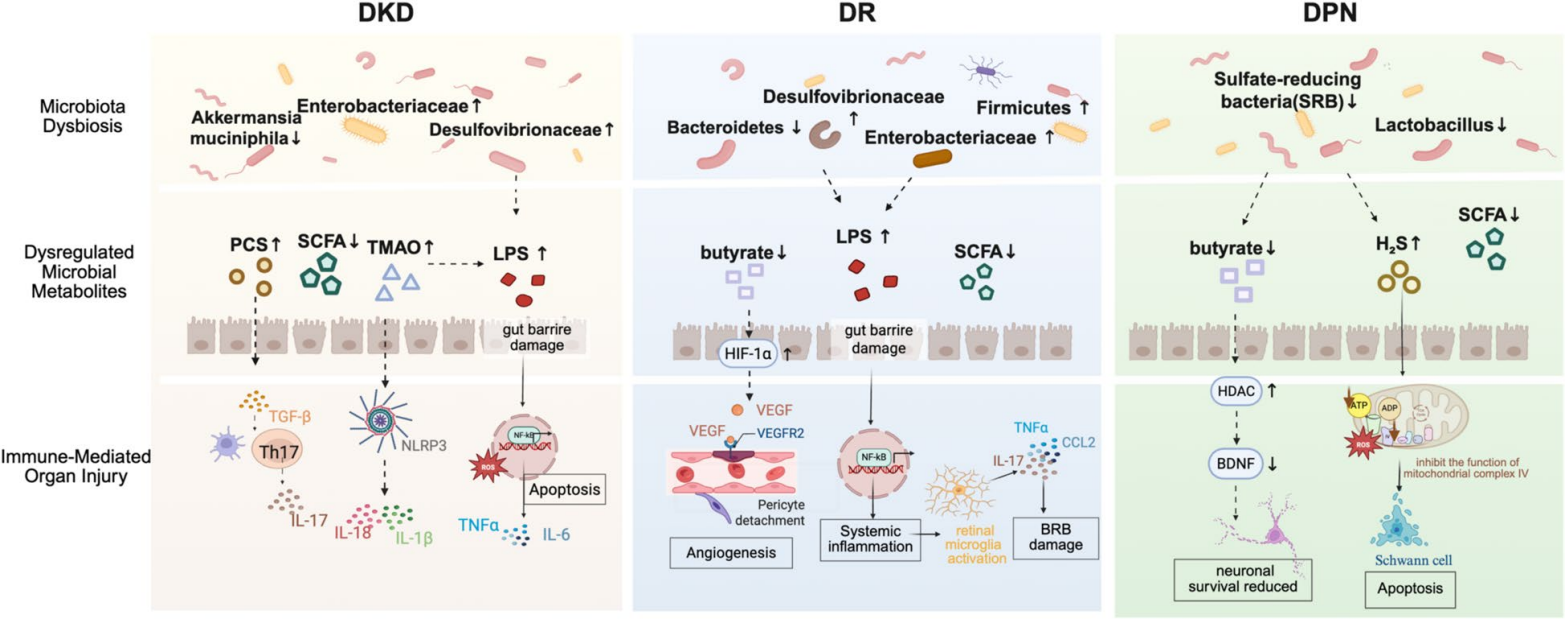
Gut microbiota dysbiosis and diabetic microvascular complications

Accumulating evidence indicates that gut microbiota dysbiosis is closely related to the initiation and progression of diabetic microvascular complications. Although these complications affect distinct target organs, emerging studies show that they share highly convergent pathogenic mechanisms. These mechanisms can be organized into three interrelated levels: alterations in gut microbial composition, dysregulation of microbiota-derived metabolites, and downstream immune and pathological effects. (Fig. 2).

#### Alterations in gut microbiota composition

The imbalance of gut microbiota composition is the most commonly reported feature of diabetic microvascular complications. In patients with DKD, the magnitude of protective bacteria such as *Akkermansia muciniphila* is markedly reduced, while endotoxin-producing taxa including Enterobacteriaceae and Desulfovibrionaceae are increased significantly [48]. Similar disorder patterns are also observed in DR, manifested as a decrease in gut microbial diversity, a decreased relative abundance of Bacteroidetes, and an increased Firmicutes-to-Bacteroidetes (F/B) ratio [49]. Moreover, inflammation-associated genera such as *Bacteroides* and *Megamonas* are progressively enriched as DR enters the proliferative stages [50].In DPN, alterations in gut microbiota are also very significant, the abundance of beneficial genera such as *Lactobacillus* is decreased and the abundance of potentially pathogenic taxa including *Prevotella* is increased [51]. Overall, these compositional changes reflect a common pattern characterized by loss of barrier-protective and SCFA-producing bacteria while expansion of Gram-negative and pro-inflammatory taxa. This imbalance creates a permissive environment for endotoxin accumulation, metabolic dysregulation, and impaired gut barrier integrity.

#### Dysregulation of microbiota-derived metabolites

Beyond compositional changes, dysbiosis-induced alterations in microbial metabolites play an important role in driving microvascular injury. In DKD, gut microbiota dysbiosis leads to excessive production of acetate, which leads to an increase in acetic level in circulation [52]. Excess acetate activates the intrarenal renin-angiotensin system, thereby inducing renal vasoconstriction, glomerular hyperfiltration, inflammatory signaling, profibrotic factor release, extracellular matrix deposition, and podocyte structural damage, ultimately accelerating DKD progression [53]. Uremic toxins derived from microbial metabolism, including trimethylamine N-oxide (TMAO) and p-cresol sulfate (PCS), also accumulate under gut microbiota dysbiosis conditions. TMAO promotes renal inflammation and fibrosis through activation of the NLRP3 inflammasome and then releasing IL-1β and IL-18, while PCS upregulates pro-inflammatory cytokines and TGF-β1 expression, exacerbating tubular injury and oxidative stress, which is demonstrated in DKD models [54, 55].In DR, gut microbiota dysbiosis will lead to a synthesis of SCFA, particularly butyrate. Butyrate deficiency disrupts retinal metabolic homeostasis by preventing efficient degradation of hypoxia-inducible factor-1α (HIF-1α), which will lead to a sustained activation of VEGF signaling and pathological retinal neovascularization [56]. Supplementing butyrate has been proven to restore gut microbial balance, characterized by reduced abundance of pro-inflammatory genera such as *Escherichia-Shigella* and *Enterococcus*, enrichment of beneficial taxa including *Lactobacillus* and *Bifidobacterium*, and reduction of retinal damage in diabetic models [57]. In DPN, dysbiosis-associated metabolic alterations include increased levels of branched-chain amino acids and hydrogen sulfide (H₂S). Excessive H₂S production driven by overgrowth of sulfate-reducing bacteria will inhibit mitochondrial complex IV, reduce ATP synthesis, and increase oxidative stress, eventually leading to Schwann cell apoptosis [58]. At the same time, depletion of SCFAs (especially butyrate) impairs histone deacetylase (HDAC) inhibition, suppresses expression of neurotrophic factors such as brain-derived neurotrophic factor (BDNF), and do harm to neuronal survival and axonal regeneration [59, 60]. Altered bile acid metabolism and accumulation of neurotoxic secondary bile acids further disrupt neuronal energy metabolism and axonal transport, thus contributing to DPN progression [61].

#### Immune and pathological processes mediating dysbiosis-induced organ injury

Gut microbiota dysbiosis and associated metabolic disturbances jointly drive immune and inflammatory signaling pathways implicated in diabetic microvascular complications. Increased abundance of Gram-negative bacteria enhances LPS release, which compromises intestinal barrier integrity and enable endotoxins enter into systemic circulation. LPS in the circulation activates the Toll-like receptor 4 (TLR4)-NF-κB signaling pathway, causing chronic low-grade inflammation and excessive production of pro-inflammatory cytokines such as IL-6, TNF-α, and IL-17 [62, 63].

In DKD, sustained activation of inflammatory and oxidative stress pathways upregulates the expression NADPH oxidase, resulting in excessive generation of ROS, glomerular basement membrane thickening, and interstitial fibrosis [64]. In DR, systemic inflammation induces a pro-inflammatory phenotype in retinal microglia, promoting neuroinflammation, blood-retinal barrier disruption, and neuronal degeneration [65, 66]. In DPN, the activation of TLR4-NF-κB axis and microbiota-derived kynurenine signaling stimulate spinal glial cells, exacerbate neuropathic pain hypersensitivity, and accelerate peripheral nerve damage [66, 67].

Overall, these findings indicate that gut microbiota dysbiosis contributes to diabetic microvascular complications through a shared immune-inflammatory and oxidative stress framework, while vulnerability of organ specificity determines the distinct pathological manifestations observed in the kidney, retina, and peripheral nerves.

## TCM modulates gut microbiota to alleviate diabetic microvascular complications

The gut microbiota plays a central role in maintaining host metabolic and immune homeostasis. Recent research has indicated that TCM could modulate the composition and function of intestinal microbiota, thereby improving host metabolic status and immune responses [68]. Through this close interaction, TCM exerts its pharmacological effects partly via microbiota-mediated transformation and metabolic regulation. By modulating gut microbiota composition [69], promoting the production of beneficial metabolites such as SCFA [70] and improving intestinal barrier integrity [71], TCM can alleviate the progression of microvascular complications in DR and DKD at multiple levels. Table 1 systematically summarises representative traditional Chinese medicine (TCM) interventions that alleviate diabetic microvascular complications by modulating the gut microbiota.

**Table 1:** Summary of TCM in regulating the gut microbiota

TCM	Components/Formulations	Microvascular complication	Research object	Changes in gut microbiota/Metabolites	Mechanism of action	Reference
Huangkui Capsule (HKC)	*Abelmoschus Manihot* extract	DKD	NOD/LtJ mice	↑ Lachnospiraceae, *Roseburia*; ↓ BCAA	Gut microbiota–metabolite axis modulates inflammation	[73]
Ramulus Mori (Sangzhi)	Mori alkaloids (SZ-A)	DKD	HFD/STZ induced C57BL/6 J mice	↑*Bacteroides*, *Dubosiella*, *Alistipes*, *Clostridium*, *Faecalibaculum*, *Lactobacillus*; ↑ 2-methylhippuric acid, 12-MTA	Improves gut microbiota disorder	[76]
Yi-Shen-Hua-Shi granule	*Panax ginseng*, *Astragalus membranaceus*, *Altractylodes macrocephala*, *Poria cocos*, *Alisma orientale*, etc	DKD	HFD/STZ induced SD rat	↑ *Lactobacillus*, *Lactobacillus murinus*; ↓ *Prevotella UCG_001*; ↓ F/B ratio; ↓ Phosphatidylethanolamine	Modulates lipid and amino acid metabolism via gut microbiota	[77]
Curcuma kwangsiensis	Germacrone	DKD	db/db mice	↓ *Serratia marcescens*, *Lactobacillus iners*; ↑ oleic acid, lithocholic acid; ↓ stearic aci	Alterate gut flora abundance, modulate endogenous metabolites	[78]
QiDiTangShen granules	*R. glutinosa*, *Astragalus propinquus, Euryale ferox*, *Cornus officinalis*, etc	DKD	db/db mice	↑ *Alloprevotella*; ↓ TCA, DCA, β-MCA, Tβ-MCA	Regulates gut microbiota–bile acid axis	[80]
Qikui granule	Astragali Radix, Abelmoschi Corolla, Polygoni Multiflori Radix Praeparata	DKD	db/db mice	↑*Candidatus_Arthromitus*, ↑*Lactobacillus*, *Bifidobacterium*, *Dubosiella*; ↓ PC, LPC, LPE	Regulates glycerophospholipid metabolism; suppresses PI3K-AKT signaling pathway	[89]

TCM	Components/Formulations	Microvascular complication	Research object	Changes in gut microbiota/Metabolites	Mechanism of action	Reference
*Astragalus membranaceus* and *Salvia miltiorrhiza*	*Astragalus membranaceus* and *Salvia miltiorrhiza*	DKD	HFD/STZ induced SD rat	↑ *Akkermansia muciniphila*; ↓ F/B ratio	Enrich *Akkermansia muciniphila* to enhance intestinal barrier	[84]
Punicalagin	Punicalagin	DKD	HFD induced C57BL/6 J mice	↑ SCFAs; ↑ *Eubacterium coprostanoligenes*, Lachnospiraceae	Enhances SCFA production and intestinal barrier	[90]
Jikan Mingmu Formula	*Berberis kansuensis* Schneid., *Carthamus tinctorius* L., etc	DR	STZ-induced SD rats	↑ *Lactobacillus*; ↓ *Helicobacter*, *Parasutterella*	Restores microbial balance, reduces inflammation	[91]
TWMM granule	*Astragalus membranaceus* (Fisch.) Bunge., *Leonurus japonicus* Houtt., etc	DR	STZ-induced C57BL/6 J mice	↑ Firmicutes; ↓ Bacteroidetes	Alter gut microbiota composition	[92]
ACHP	*Angelica sinensis*, *Carthamus tinctorius*	DR	STZ-induced C57BL/6 J mice	↑ *Lactobacillus*; ↓ *Dubosiella*, *Candidatus Saccharimonas*	“Gut-eye-brain” axis, transcriptomic–metabolomic integration	[83]
Bushen Huoxue Prescripti	*Salvia miltiorrhiza Bge*., *Pueraria montana* (Lour.) Merr., etc	DR	db/db mice	↑ Actinobacteria; ↓ Firmicutes	Bile acid metabolism modulation, anti-inflammatory	[93]

TCM	Components/Formulations	Microvascular complication	Research object	Changes in Gut Microbiota/Metabolites	Mechanism of action	Reference
Luo Tong formula	Radix Rhei Et Rhizome, Persicae Semen, *Panax notoginseng*, Hirudo	DR	STZ-injected SD rats	↑ Firmicutes; ↓ Bacteroidetes; ↑ ZO-1, occludin, claudin-5, VE-cadherin	Enhances cell junction proteins to protect BRB	[85]
Compound Qilian tablets	*Astragalus membranaceus*, *Rehmannia glutinosa*, *Coptis chinensis*, *Panax notoginseng*	DR	ZDF (fa/fa) and ZDF (fa/+) rats	↑ *Akkermansia*, *Clostridium_sensu_stricto_1*; ↓ *Negativibacillus*; ↑ L-glutamine	Maintains gut barrier integrity	[94]
Quercetin	Quercetin (flavonoid)	DPN	STZ-induced SD rats	↑Actinobacteria; ↓Porphyromonadaceae, Oxalobacteraceae	Antioxidant modulation via ROS-sensitive microbes	[95]
Jinmaitong	*Ligustrum lucidum* Ait., *Lycium barbarum* L., etc	DPN	STZ-induced SD rats	↑ Actinobacteria, *Proteobacteria*; ↓ Porphyromonadaceae	Reshape gut microbiota composition and reduces neuroinflammation	[72]
Danggui Sini Decoction	*Angelica sinensis*,Cinna momum cassia,etc	DPN	/	↑ *Allobaculum*, *Dubosiella*; improved SCFAs	Alter gut microbiota composition	[96]
HGWD	*Angelica sinensis*, *Cinnamomumcassia*, etc	DPN	db/db mice	↑ *Lactobacillus*; ↓ *Desulfovibio*	Sphingolipid metabolism enhancement, nerve repair	[97]

### TCM modulates gut microbiota composition to alleviate diabetic microvascular complications

TCM has emerged as a promising therapeutic approach for diabetic microvascular complications. Accumulating evidence suggests that its beneficial effects are largely mediated through modulation of the gut microbiota, which in turn influences host metabolic and immune pathways. By reshaping microbial homeostasis, TCM can mitigate chronic low-grade inflammation and ameliorate microvascular injury in target organs. These multi-target actions highlight the potential of TCM as a complementary strategy for managing diabetes-associated microvascular disorders.

Jinmaitong has been shown to ameliorate DKD in streptozotocin (STZ)-induced diabetic rat models by significantly enriching phyla such as Actinobacteria and Proteobacteria, and class-level Actinobacteria, while reducing the abundance of f_Porphyromonadaceae and *g_Prevotella*. This restructuring of the gut microbial community appears to contribute to the alleviation of neuropathic symptoms through the regulation of gut-derived neuroimmune interactions. Notably, this microbial modulation was accompanied by improvements in neuroprotective pathways involving neuregulin-1 signaling, suggesting that the therapeutic benefits of Jinmaitong are closely linked to both microbial remodeling and neuronal repair. Jun Xie et al. [72] demonstrated that Jinmaitong reshaped the gut microbiota in DPN rats by promoting beneficial microbial taxa while suppressing potentially pathogenic bacteria, thereby ameliorating neuropathic phenotypes and offering a promising strategy for targeting gut-nerve axis dysfunction in diabetic neuropathy. Additionally, Shi et al. [73] conducted a study on Huangkui Capsule (HKC) and demonstrated that it improved DKD outcomes by modulating the gut microbiota composition in non-obese diabetic (NOD) mice. Specifically, treatment with HKC significantly increased the relative abundance of beneficial genera including Lachnospiraceae_NK4A136_group, *Muribaculum*, *Faecalitalea*, *Parasutterella*, *Roseburia*, Lachnospiraceae_FCS020_group, and *Odoribacter*. These microbial shifts were closely associated with altered host metabolic profiles, characterized by reduced levels of branched-chain amino acids (valine, leucine, and isoleucine) and elevated levels of cis-7-hexadecenoic acid, a metabolite with anti-inflammatory potential. These findings suggest that HKC exerts renoprotective effects through gut microbiota-mediated metabolic modulation, highlighting its potential to restore gut-kidney axis homeostasis in DKD.

Overall, TCM alleviates microvascular injury by enriching probiotics, reducing pathogenic bacteria, and restoring microbial homeostasis. In the context of increasingly recognized inter-organ axes-including the gut-retina, gut-kidney, and gut-nerve, reconstructing gut microbial communities has emerged as a vital target in TCM-based approaches to treat diabetic microvascular complications.

### TCM modulates gut microbiota-derived metabolites to alleviate diabetic microvascular complications

In healthy hosts, the gut microbiota ferments dietary components via glycolysis and other metabolic pathways to produce key metabolites such as SCFAs, which are essential for maintaining intestinal epithelial homeostasis and regulating systemic metabolism [74]. Increasing evidence suggests that TCM can regulate the gut microbiota and related metabolites to intervene in the course of diabetic microvascular complications.

You et al.[75] found that Qikui Granule, a multi-component TCM formula containing *Astragalus membranaceus* and *Abelmoschus manihot*, significantly altered the levels of glycerophospholipid metabolites such as phosphatidylcholine (PC), lysophosphatidylcholine (LPC), and lysophosphatidylethanolamine (LPE), thereby contributing to the amelioration of glomerular injury in DKD mice. Liu et al.’s [76] study revealed that increasing microbial-derived SCFA levels is closely linked to enhanced epithelial barrier integrity and reduced renal inflammation in diabetic models, which aligns with the observed effects of Ramulus Mori Alkaloids in high-fat diet (HFD) and STZ-induced DKD mice. Additionally, Yi-Shen-Hua-Shi Granule [77], a traditional decoction targeting “spleen and kidney deficiency with damp-heat”, exerted renoprotective effects through modulation of the gut microbiota-host metabolism axis. The increase in *Lactobacillus murinus* and decrease in *Prevotella UCG_001* abundance led to improved phosphatidylethanolamine metabolism and suppression of oxidative stress and chronic inflammation. Furthermore, Wang et al. [78] conducted a study on the lipidomic effects of Germacrone and found that it significantly reshaped fatty acid and bile acid metabolic profiles in db/db mice, which correlated with improved renal pathology and function. Wang et al. discovered that the modulation of the gut microbiota by TCM can significantly influence host lipid and bile acid metabolism, thus offering new therapeutic insights for microvascular complications of diabetes. It is also discovered that butyrate supplementation in STZ-induced diabetic mice improved retinal health. It enhanced intestinal barrier integrity, increasing tight junction proteins (ZO-1, Occludin) and restoring beneficial bacteria like *norank_f_Muribaculaceae*, *Ileibacterium*, and *Dubosiella*, which were positively correlated with butyrate and other SCFAs. These SCFAs, including butyric acid, 4-methylvaleric acid, and caproic acid, contributed to anti-inflammatory and neuroprotective effects, improving retinal function [79]. In their study QiDiTangShen granules [80] have been demonstrated that modulation of the gut microbiota-bile acid axis was closely associated with renal protection. Treatment of QiDiTangShen granules led to a reduction in the abundance of *Lactobacillus*, *Bacteroides*, *Roseburia*, and *Lachnospiraceae_NK4A136_group*, alongside an increase in *Alloprevotella*. These microbial shifts were accompanied by significantly decreased levels of bile acids including TCA, DCA, β-MCA, and Tβ-MCA, suggesting that improved bile acid profiles contributed to the observed renoprotective effects.

These studies suggest that TCM may attenuate diabetes-associated microvascular injury by targeting metabolites produced by the gut microbiota. As mediators of inter-organ communication along the gut-kidney and gut–retina axes, these metabolites are emerging as novel therapeutic targets for TCM-based interventions in diabetic microvascular complications.

### TCM repairs intestinal barrier function to alleviate diabetic microvascular complications

The intestinal barrier is important in containing gut homeostasis, restricting the translocation of endotoxins into the circulation, and regulating systemic inflammation[81].Tight junction proteins are essential structural components of the epithelial barrier. Disruption of barrier function can lead to increased intestinal permeability (“leaky gut”), allowing endotoxins like LPS to enter the bloodstream. This promotes chronic low-grade inflammation, which further exacerbates the progression of diabetic microvascular complications. Increasing evidence suggests that TCM can regulate the gut microbiota and related metabolites to intervene in the course of diabetic microvascular complications [82]. Increasing evidence suggests that TCM can improve complications such as DR and DKD by repairing the gut barrier function and supporting mucosal immune stability.

Shen et al. [83] discovered that the Angelica sinensis-Carthamus tinctorius herb pair (ACHP) exerts a multifaceted protective effect on intestinal integrity in DR mouse models. ACHP administration enhanced the abundance of beneficial bacteria like *Lactobacillus*, while suppressing harmful genera including *Dubosiella*, *Faecalibaculum*, *Achromobacter*, and *Pseudomonas*, thereby restoring microbial balance. ACHP upregulated the expression of epithelial tight junction proteins ZO-1 and Occludin, as well as the mucin-secreting marker MUC-2, reinforcing both the epithelial and mucus barriers. It is also reported that ACHP significantly reduced serum LPS concentrations, thereby dampening systemic inflammation through inhibition of TLR4/NF-κB signaling. Shen et al. [84] showed that the combination of *Astragalus membranaceus* and *Salvia miltiorrhiza* ameliorated renal histopathological changes and improved colonic permeability in DKD rats. The treatment modulated the gut microbiota by lowering the Firmicutes/Bacteroidetes ratio and selectively enriching *Akkermansia muciniphila*, a mucin-degrading probiotic known to promote barrier repair. These microbial alterations were linked to improved epithelial alignment and increased goblet cell numbers, suggesting enhanced mucus secretion and epithelial renewal. Furthermore, Di et al. [85] conducted a study on Luo Tong formula (LTF) in STZ-induced DR rats and demonstrated that LTF significantly upregulated tight junction proteins ZO-1, Occludin, and Claudin-5, along with adherens junction protein VE-cadherin in colon tissues. These changes were associated with reduced intestinal permeability and lower systemic LPS levels, thereby mitigating inflammation-related damage to the blood-retinal barrier.

These findings suggest that TCM formulations exert multi-target regulatory effects on intestinal barrier function by modulating microbiota composition, reducing endotoxin levels, and enhancing the expression of epithelial junctional proteins. Such interventions reduce pro-inflammatory cytokines like TNF-α and IL-6, attenuate microvascular endothelial activation, and alleviate tissue injury in target organs like the kidney and retina. As the gut-retina and gut-kidney axes continue to gain recognition, stabilizing intestinal barrier function represents a promising therapeutic strategy for the prevention and treatment of diabetic microvascular complications.

### Causal evidence supporting gut microbiota–dependent mechanisms of TCM interventions

Accumulating evidence has shown strong associations between TCM administration, gut microbiota modulation, and improvement of diabetic phenotypes and microvascular complication phenotypes, however, the causality of gut microbiota alterations in mediating TCM-induced therapeutic effects remains to be fully addressed. To address this issue, a growing number of studies have employed antibiotic-treated (ABX) model and subsequent fecal microbiota transplantation (FMT) strategies to directly test causality. In various diabetic models, the use of broad-spectrum antibiotics cleared the intestinal microbiota which significantly weakened or completely eliminated the therapeutic effects of TCM formulations, indicating that an intact microbial ecosystem is necessary for the efficacy of TCM. For example, Jinlida was shown to ameliorate the progression of DKD through microbiota-dependent production of pyridoxamine, while these effects were reduced and accompanied by loss of suppression of the AGEs/RAGE and TGF-β pathways in the ABX model [86]. Fan et al. reported that echinacoside (ECH) markedly ameliorated metabolic dysfunction in T2DM mice, but the effects of improving hyperglycemia and inflammation were reduced in the ABX model, supporting the notion that gut microbiota integrity is necessary for ECH-mediated therapeutic benefits [87]. In addition to establishing the necessity of the gut microbiota for TCM efficacy, FMT-based studies suggest that TCM-induced microbial alterations are functionally active and partially transferable. For instance, FMT of microbiota from mulberry leaf water extract–treated donors into pseudo-germ-free diabetic mice transferred improvements in glucose homeostasis and white adipose browning via SCFA-activated AMPK/SIRT1/PGC-1α signaling, supporting functional transmissibility of TCM-modulated communities [76]. Similarly, FTZ polysaccharide-associated microbiota transferred via FMT restored intestinal barrier integrity and ameliorated renal injury in DKD models, indicating that microbiota reconfiguration following TCM intervention can drive host tissue protection [88].

Collectively, evidence from ABX models and FMT studies moves current findings beyond association and supports a causal contribution of the gut microbiota to TCM-induced therapeutic effects. Antibiotic-mediated microbiota depletion highlights the necessity of an intact microbial ecosystem, meanwhile FMT experiments suggest that TCM-modulated microbial communities can transmit protective phenotypes. Nevertheless, causal evidence remains uneven across formulations and disease contexts, underscoring the need for deeper mechanistic resolution in future studies.

## Bioactive constituents of Chinese herbal medicine in gut microbiota regulation

### Macromolecular components of TCM in modulating gut microbiota

Macromolecular bioactive compounds from TCM broadly refers to chemical constituents of TCM with relatively large molecular weights that are difficult to digest or absorb in the host's small intestine [98]. These include polysaccharides, prototypes of saponins (often glycosides), and certain functional peptide segments [99, 100]. These substances cannot be directly absorbed in the small intestine and reach the colon largely intact, where they act as substrates for microbial fermentation or modulate gut microbial composition and function [101], thereby indirectly influence host metabolism and immunity through reshaping microbial community structure and metabolic profiles [102].

TCM polysaccharides are typical natural macromolecules with high molecular weight [103]. Due to their large size and complex branched structures, they cannot be directly absorbed into the bloodstream by the host digestive system. Instead, they reach the colon where they are broken down as food sources and nutritional substrates for the gut microbiota [104]. The “prebiotic effect” of polysaccharides represents one of the most well-established aspects of TCM-microbiome research [105]. Acting as nutritional substrates, polysaccharides undergo selective fermentation by specific microbial populations, thereby promoting the proliferation of probiotic bacteria such as *Bifidobacteria*, *Lactobacillus*, and *Akkermansia* while inhibiting the excessive expansion of certain harmful microbial groups [106]. For instance, Astragalus polysaccharides (APS) increase *Akkermansia* and *Lactobacillus* abundance, improving insulin resistance and inflammation in mice. Inulin-type polysaccharides have also been shown in human studies to elevate bifidobacteria counts and reduce glycated hemoglobin (HbA1c) [107], a key biomarker reflecting long-term glucose control and metabolic homeostasis in diabetic patients. SCFAs produced during polysaccharide fermentation, such as acetate, propionate, and butyrate, act as signaling molecules that enhance host insulin sensitivity and mitigate systemic inflammatory responses through pathways including GPR41/43 receptors [108]. Zhang et al. found that Lycium barbarum polysaccharides (LBP) improved gut homeostasis in diabetic mice by elevating butyrate levels and strengthening the intestinal barrier [109]. Although previous work suggests that molecular weight and glycosidic bond types influence microbial fermentation products, few systematic studies have directly correlated structural features with microbial responses. Future approaches combining “chemical structure analysis” and “microbial function prediction” may reveal clearer “prebiotic effect” relationships.

Large-molecule saponins from Chinese herbal medicines are often described as having a potential “bidirectional regulation” with the gut microbiota [110]. These compounds, such as ginsenosides, generally exist as glycosides with high molecular weight and strong polarity, which limits their intestinal absorption and results in low plasma concentrations after oral administration. Increasing evidence indicates that gut microbiota plays a crucial role in their bioactivation: enzymes like β-glucosidase can deglycosylate parent saponins into more absorbable aglycones or secondary metabolites with enhanced pharmacological activity. For instance, ginsenoside Rb1 is progressively transformed by gut microbiota into compound K, the major circulating form, which shows improved bioavailability and metabolic benefits compared with its precursor [111]. Similar processes occur with other saponins-for example, glycyrrhizic acid is converted into glycyrrhetinic acid, and steroidal saponins into more hydrophobic sapogenols-resulting in higher systemic exposure and distinct anti-inflammatory or insulin-sensitizing effects [112]. Moreover, these metabolites are not merely passively absorbed but can selectively reshape gut microbiota composition. Interestingly, these metabolites can also exert feedback effects on the gut microbiota. For example, compound K has been reported to enrich beneficial taxa such as *Akkermansia muciniphila* and to reduce LPS-producing bacteria, thereby improving gut barrier integrity and attenuating chronic inflammation [113, 114]. Collectively, saponins and their microbiota-derived metabolites form a dynamic regulatory loop-, where microbial metabolism activates saponins into bioactive forms and these metabolites in turn selectively optimize the gut microbial ecosystem.

### Small-molecule components of TCM in modulating gut microbiota

Bioactive small molecules derived from TCM generally refer to chemical constituents with molecular weights below 1000 Da with high bioavailability. Compared with macromolecules that can act as substrates for microbial fermentation, small molecules may more directly serve as signaling mediators in host-microbiome interactions [115]. They directly influence microbial composition and host metabolic pathways, exerting pharmacological effects with higher specificity and more rapid onset. Representative compounds such as quercetin, a naturally occurring flavonoid, have demonstrated such regulatory activity. Xie et al. [95] discovered that quercetin exerted neuroprotective effects in DKD models by modulating gut microbiota composition. It significantly suppressed inflammation-associated bacteria, such as *Klebsiella*, *Oxaloptera*, and members of the families Porphyromonadaceae and Oxalobacteraceae, while enriching beneficial taxa, including Actinobacteria and other SCFA-producing microbes. These microbial shifts were associated with reduced ROS production and modulation of antioxidant pathways, including glutathione metabolism and NADPH redox balance. Such improvements contributed to the amelioration of DPN symptoms like sensory dysfunction and delayed nerve conduction.

Similarly, other TCM-derived small molecules display distinct microbiota-modulating properties. Phenolic compounds can selectively inhibit pathogenic bacterial overgrowth by disrupting membrane integrity, inhibiting key enzymes such as DNA gyrase, or modulating oxidative stress responses [116]. They are also metabolized by gut microbiota into secondary metabolites, which further reshape SCFA and bile acid metabolic profiles [117]. Alkaloids display both direct antibacterial activity and dual host-microbiome effects [118]. Berberine, for instance, inhibits Gram-negative bacteria and opportunistic pathogens while simultaneously stimulating mucus secretion and enhancing MUC2 expression [119]. This creates a favorable niche for *Akkermansia muciniphila* and synergistically activates the AMPK pathway to promote metabolic homeostasis [120]. Terpenoid compounds also exhibit distinct microbiota-modulating properties. Artemisinin improves glucose homeostasis while altering microbial composition, whereas polyphenolic terpenoids such as rosmarinic acid alleviate intestinal inflammation, repair tight junctions, and indirectly improve host metabolism by enhancing SCFA-producing taxa [121]. Organic acids (e.g., citric acid, succinic acid) mainly act as environmental regulators [122]. By serving as microbial substrates and energy sources [123], they directly alter intestinal pH and nutrient availability, thereby supporting acid-tolerant probiotics while suppressing harmful bacteria. Functional peptides represent pivotal mediators within the “peptide-microbiota-host” signaling axis. On one hand, they can serve as nitrogen sources for microbes; on the other, they exert selective antibacterial activity similar to antimicrobial peptides. Additionally, they can be sensed by intestinal epithelial cells, stimulating the release of gut-derived hormones such as GLP-1 [124], and thereby indirectly modulating microbiota remodeling through metabolic and immune pathways. Table 2 systematically summarises representative TCM-derived macromolecular and small-molecule components that modulate the gut microbiota and related microbial metabolites.

**Table 2:** Summary of major TCM-derived components modulating gut microbiota and microbial metabolites

Compound/Component	TCM source	Related mechanisms	Reference
Astragalus polysaccharides (APS)	*Astragalus membranaceus*	↑ *Akkermansia*, ↑ *Lactobacillus*, ↑ SCFAs, ↓ inflammation, improvement of insulin resistance and metabolic inflammation	[106]
Inulin-type polysaccharides	*Cichorium intybus* (chicory root), *Helianthus tuberosus* (Jerusalem artichoke)	↑ *Bifidobacterium*, ↑ SCFAs, ↓ HbA1c, improvement of glycemic control in humans	[107]
Lycium barbarum polysaccharides (LBP)	*Lycium barbarum*	↑ SCFA-producing bacteria, ↑ butyrate, strengthened intestinal barrier, improvement of gut homeostasis and metabolic status	[109]
Compound K (microbial metabolite)	Microbiota-derived from ginsenosides	↑ *Akkermansia muciniphila*, ↓ LPS-producing bacteria, ↓ LPS, improved gut barrier integrity, anti-inflammatory and metabolic benefits	[113]
Glycyrrhizic acid	*Glycyrrhiza uralensis*	conversion to glycyrrhetinic acid, enhanced systemic exposure after microbial metabolism	[112]

Compound/Component	TCM source	related mechanisms	Ref
Quercetin	*Sophora japonica* L	↓ *Klebsiella*, ↓ *Oxaloptera*, ↓ Porphyromonadaceae, ↑ Actinobacteria, ↑ SCFA-producing bacteria, ↓ ROS-related metabolites, ↑ antioxidant pathways, ↓ inflammation, neuroprotection, improved nerve function in DPN	[95]
Berberine	*Coptis chinensis*	↓ Gram-negative bacteria, ↑ *Akkermansia muciniphila*, ↓ LPS, altered SCFAs, ↑ MUC2 expression, improved mucus barrier, AMPK activation, metabolic homeostasis	[119]
Artemisinin	*Artemisia annua*	Altered microbial composition, improved glucose homeostasis	[121]
Citric, succinic acid	Endogenous/plant-derived	↑ acid-tolerant probiotics, ↓ harmful bacteria, altered intestinal pH and nutrient availability	[122]

### Exosome-like nanovesicles from TCM in modulating gut microbiota

In recent years, PELNs and TCM-derived exosome-like nanovesicles (TCM-ELNs) have been increasingly recognized as bioactive mediators [125]. These nanovesicles can directly interact with microbial and host cells, modulating the “gut-microbiota-host” network through multiple complementary mechanisms [126]. Their effects can be categorized into three major pathways:

- 1) Direct microbiota modulation. PELNs/TCM‑ELNs comprise a variety of bioactive components, including membrane lipids, proteins and enzymes, small‑molecule metabolites and nucleic acids, which act synergistically to form a natural system for modulating the gut microbiota [127]. These vesicles can interact with or be internalized by specific bacterial strains, thereby directly altering microbial composition and metabolic activity [128]. For example, vesicles derived from ginger and grape sources have been shown in animal models to increase beneficial genera such as *Lactobacillus* and to reduce certain pro‑inflammatory taxa such as Enterobacteriaceae [129]. Citrus exosome‑like nanoparticles have been demonstrated in high‑fat diet‑induced gut dysbiosis models to lower the abundance of potential pathogens such as *Prerotellamassilia timonensis*, increase beneficial taxa including *Acetatifactor muris* and *Phocaeicola sartorii*, and elevate SCFA levels, thereby restoring microbial homeostasis and metabolic function [130]. Honeysuckle-derived nanovesicles were shown to markedly reshape gut microbial composition in DSS-induced colitis models by increasing short-chain fatty acid production, reprogramming bile acid metabolism, and restoring of immune homeostasis [131]. Edible exosome-like nanoparticles derived from *Portulaca oleracea* L. alleviated DSS-induced intestinal inflammation by selectively promoting beneficial bacteria, such as *Lactobacillus reuteri,* and enhancing microbiota-derived indole metabolites. The cargo lipids and metabolites in these vesicles may influence bacterial membrane structure and metabolic pathways, playing a pivotal role in this regulation [132]. Overall, the multifaceted composition of PELNs/TCM‑ELNs endows them with the capacity to directly regulate gut microbial ecology and metabolic output.
- 2) Epithelial barrier modulation. PELNs/TCM-ELNs can be internalized by intestinal epithelial cells, repairing the barrier, and reducing LPS leakage [133]. Improved barrier function subsequently feeds back on the microbiota by altering the local metabolite milieu (e.g., increased SCFAs, adjusted bile acid ratios, reduced LPS/TMAO), while also modulating host metabolism and inflammation through AMPK, PPAR, and NF-κB/NLRP3 pathways [134]. For example, vesicles derived from ginger or dried tangerine peel improved barrier integrity and restored metabolic pathways in animal models, suggesting the existence of an “epithelium‑microbiota‑metabolism” feedback loop [135].
- 3) Cross-kingdom miRNA signaling. Plant miRNAs carried by PELNs/TCM-ELNs can enter microbes or host immune cells to regulate gene expression. For instance, osa-miR164d from ginger vesicles modulates TAB1/NF-κB signaling in macrophages, promoting an anti-inflammatory phenotype [136]. Similarly, bol-miR159 has been shown to alter gut microbial composition in mice [137]. These findings demonstrate that plant miRNAs act as functional, traceable regulators bridging microbial and host immune pathways.

In summary,

PELNs/TCM-ELNs influence the gut ecosystem and host physiology through three complementary mechanisms: direct microbiota modulation, epithelial barrier repair, and cross-kingdom miRNA regulation. This mechanistic framework not only explains the metabolic and anti-inflammatory effects observed in animal studies but also provides a basis for future validation in complex disease models, including diabetic microvascular complications. Table 3 systematically summarises the mechanisms of PELNs/TCM-ELNs in modulating the gut microbiota.

**Table 3:** Mechanisms of PELNs/TCM-ELNs in modulating gut microbiota

Exosome-like vesicles	Plant/TCM source	Mechanism of action on gut microbiota	Reference
Grape-derived exosome-like nanoparticles	*Vitis vinifera*	↑ *Lactobacillus*, ↓ Enterobacteriaceae, ↓ pro-inflammatory taxa	[129]
Citrus exosome-like nanoparticles	*Citrus* spp.	↓ *Prevotellamassilia timonensis*, ↑ *Acetatifactor muris*, ↑ *Phocaeicola sartorii*, ↑ SCFAs, restoration of microbial homeostasis and metabolic function	[130]
Honeysuckle-derived nanovesicles	*Lonicera japonica* Thunb	Altered gut microbial composition, ↑ SCFAs, reprogrammed bile acid metabolism, restoration of immune homeostasis	[131]
Edible exosome-like nanoparticles	*Portulaca oleracea* L	↑ *Lactobacillus reuteri*, ↑ microbiota-derived indole metabolites	[132]
Tangerine Peel Exosome-like Nanoparticles	*Citrus reticulata* Blanco	↑ tight junction proteins(ZO-1, occludin) ↓ intestinal permeability	[135]
Ginger-derived exosome-like nanoparticles	*Zingiber officinale* Roscoe	↑ osa-miR164d-mediated TAB1/NF-κB inhibition in macrophages and ↑ anti-inflammatory phenotype, bol-miR159-mediated alteration of gut microbial composition	[136]

## Discussion and outlook

As an “invisible organ”, the gut microbiota is involved in many physiological processes such as energy metabolism, immune regulation, and intestinal barrier maintenance [138]. Dysbiosis of the gut microbiota is considered closely associated to diabetes and its multiple chronic complications [139]. In recent years, the mechanism of TCM regulating the gut microbiota to improving host metabolic homeostasis in order to delay or prevent the development of diabetes mellitus and its microvascular complications have garnered growing scientific interest [140]. This reviews primarily summarizes the correlation between gut microbiota and the microvascular complications of diabetes, and integrate current research on how TCM regulates gut microbial communities, influencing microbial composition, endogenous metabolites, and the repair of intestinal barrier function.

Most studies focused on descriptive investigation of microbiota structure and metabolites, lacking in-depth analysis of causal relationships. Most experiments use 16-sRNA sequencing technology to focus on changes in the overall microbiota, but due to limited resolution, it is difficult to accurately identify the abundance changes and functional associations of specific species or strains [141]. To address this limitation, the resolution and accuracy of strain identification can be improved by applying metagenomics [142]. Also, combined with metabolomics, the distribution and dynamic changes of metabolites in blood and target tissues (kidney, retina) can be quantitatively analyzed by LC–MS/MS, and the association between bioavailability and pharmacological relevance can be clarified [143]. Furthermore, the application of ABX animal models and FMT straprovides powerful tools to establish causality and dissect the mechanistic links between TCM, gut microbiota, and host physiological outcomes. And cellular specificity of microbiota-derived metabolites can be revealed by accurately identifying the differences in the response of target tissue cell subsets to microbiota metabolites and the activation status of key signal pathways, and then specifically analyzing the “TCM-microbiota-host” Interaction networks to identify key functional strains and metabolic pathways [144].

The gut microbiota functions are both a target of TCM and a synthesis platform of the bioactive compounds, acting as a critical bridge linking TCM-derived compounds to host physiological effects [145]. The polysaccharides, polyphenols, and saponins from TCM can produce a variety of biologically active metabolites under the action of intestinal flora, which can further participate in multiple physiological processes such as antioxidant, anti-inflammatory, glucose and lipid regulation, and potentially affect microvascular function [146]. This bidirectional interaction mechanism suggests that microbiota-derived metabolic enzymes play a vital role in mediating the biotransformation of TCM compounds and should be carefully considered when investigating the mechanisms of TCM in treating microvascular complications of diabetes [147]. However, most of the existing studies focus on how TCM alert the structure of the intestinal microbiota and thus changes the disease phenotype, but it remains unclear how the gut microbiota transform and modify TCM components and it is still difficult to identify the specific bioactive metabolites that exert therapeutic effects via microbial conversion [148, 149]. To address this issue, multiple approaches can be employed to investigate the specific mechanisms of therapeutic effects of TCM through modulation by the gut microbiota. For example, organoid models or in vitro gut-mimicking systems can be used to further explore the interactions between gut microbes and TCM components [150, 151]. In addition, engineered bacterial strains expressing specific metabolic enzymes can be designed to verify whether they enhance the bioconversion efficiency of TCM bioactive compounds [152]. In recent years, PELNs have emerged as a novel class of bioactive agents capable of improving metabolic abnormalities [153]. For example, ginger-derived nanoparticles [154] and mung bean sprout-derived nanovesicles [155] have demonstrated protective effects in HFD-induced obesity and insulin resistance models. These findings suggest that PELNs may improve glucose metabolism by modulating metabolic pathways and inflammatory responses. However, most studies focus on overall metabolic improvements, and evidence for their effects on organ-specific pathologies associated with diabetic microvascular complications-such as proteinuria, glomerulosclerosis, or retinal vascular leakage-remains scarce.

Thus, future studies should focus on clarifying mechanistic pathways of “Chinese medicine-gut microbiota-microbial enzymes-pharmacological” effects and promoting clinical translation and validation. Identifying the metabolic pathways, key transforming enzymes, and target tissues of TCM active compounds in the gut are critical steps toward uncovering their pharmacological mechanisms [156]. With the rapid development of metagenomics, metabolomics, and AI-assisted structural prediction and functional verification technologies, this line of research is not only technically feasible but also show considerable potential for theoretical innovation and clinical translation [157]. On the other hand, to achieve clinical translation requires rigorous validation in both preclinical and clinical phases, including confirmation of the bioavailability and pharmacological activity of microbial metabolites in target tissues, as well as evaluation of their safety and inter-individual variability [158]. Addressing these key issues will deepen our understanding of the interaction network of “gut microbiota-TCM-the host”, and accelerate the development and application of precision TCM-based strategies for the treatment of diabetes and its microvascular complications.

## Conclusion

In summary, TCM exhibits promising potential and distinctive therapeutic value in mitigating diabetic microvascular complications through modulation of the gut microbiota. With research progress, these interventions may contribute to more comprehensive and effective strategies for protecting microvascular function in diabetes. Nevertheless, the intricate biological interactions between the gut microbiota and the host, as well as the clinical translation of experimental outcomes, remain insufficiently understood. Integrating TCM with emerging technologies-such as multi-omics profiling, artificial intelligence-assisted analysis, and advanced drug delivery systems-may provide innovative pathways for precision therapy. Hence, long-term and in-depth investigations are warranted to clarify underlying mechanisms and accelerate the clinical application of TCM in the management of diabetic microvascular complications.

## Data Availability

No datasets were generated or analysed during the current study.

## Abbreviations

AGEs: Advanced glycation end products
AR: Aldose reductase
TCM-ELNs: TCM-derived exosome-like nanoparticles
DR: Diabetic retinopathy
DKD: Diabetic kidney disease
DPN: Diabetic peripheral neuropathy
HFD: High-fat diet
LPS: Lipopolysaccharide
LTF: Luo Tong formula
NF-κB: Nuclear factor-κB
PC: Phosphatidylcholine
PCS: p-cresol sulfate
PKC: Protein kinase C
PELNs: Plant-derived exosome-like nanovesicles
ROS: Reactive oxygen species
SCFAs: Short-chain fatty acids
STZ: Streptozotocin
TCM: Traditional Chinese Medicine
TLR4: Toll-like receptor 4
TMAO: Trimethylamine N-oxide
